# Skin autoimmunity might be associated with increased efficacy of atezolizumab in metastatic urothelial carcinoma: a case report

**DOI:** 10.3325/cmj.2019.60.552

**Published:** 2019-12

**Authors:** Deni Rkman, Robert Likić, Marko Bebek, Milena Gnjidić, Marija Gamulin

**Affiliations:** 1 Department of Internal Medicine, University of Zagreb School of Medicine, Zagreb, Croatia; 2 Unit for Clinical Pharmacology Department of Internal Medicine, University Hospital Centre Zagreb, Zagreb, Croatia; 3 Division for Genitourinary Tumors, Department of Oncology, University Hospital Centre Zagreb, Zagreb, Croatia; 4 Department of Oncology, University of Zagreb School of Medicine, Zagreb, Croatia

## Abstract

Atezolizumab is a monoclonal antibody immune checkpoint inhibitor that binds to programmed death ligand 1 to selectively prevent its interaction with programmed cell death-1 (PD-1) and B7.1 (CD80) receptors. We present a case of a 61-year-old man with metastatic urothelial carcinoma of the right ureter and urinary bladder. After gemcitabine/cisplatin as the first-line chemotherapy and surgery, the patient received atezolizumab 1200 mg i.v. q3w. Following the first atezolizumab administration, he noted vitiligo periorally, on his hands, legs, and the scalp. The patient’s overall survival (OS) of >26 months and continuing response to atezolizumab treatment is considerably better than median OS in the SAUL study of 8.7 months (IMvigor211-like patients’ OS 10.0 months). This case indicates that increased efficacy of atezolizumab can be associated with cutaneous immune related adverse events, reflecting the known Th17 polarization of these diseases and showing that individuals with cutaneous adverse events could benefit from PD-1 checkpoint blockade in the therapy of metastatic urothelial carcinoma.

Atezolizumab is a humanized monoclonal antibody that prevents the binding of programmed death ligand 1 (PD-L1) to the programmed cell death-1 (PD-1) and B7.1 (also known as CD80) receptor. PD-L1, a transmembrane protein expressed on tumor cells, upon binding to PD-1 and CD80 reduces anti-tumor T-cell activity (1). Atezolizumab is prescribed for the therapy of triple negative, metastatic, or locally advanced breast cancer, extensive stage small cell lung cancer, non-small cell lung cancer, and urothelial carcinoma that has progressed during or after a prior platinum-based chemotherapy (2-6).

## Case report

A 61-year-old, well developed, well nourished, white male, who smoked a pack daily for 45 years but was otherwise healthy, was admitted to our department in June 2015 for recurrent hematuria, requiring transurethral resection of the urinary bladder, which revealed a urothelial papillary carcinoma G2. Multi-slice computed tomography (MSCT) scan in July 2015 demonstrated bilateral ureterovesical junction infiltration by the tumor, with right hydronephrosis and multiple pulmonary metastases (Table 1). Due to a deteriorating renal function (creatinine 564 µmol/L, blood urea nitrogen [BUN] 22.3 mmol/L), in September 2015 a right sided “JJ” catheter was placed, which led to an improvement in kidney function (creatinine 153 µmol/L, BUN 9 mmol/L). The patient had Eastern Cooperative Oncology Group (ECOG) performance status 0. From October 2015 to April 2016, six cycles of gemcitabine/cisplatin chemotherapy were administered, with a MSCT scan reevaluation after three months showing a regression of pulmonary metastases and a control MSCT scan after chemotherapy showing stable disease.

**Table 1:** Patient care timeline

Time	Event
June 2015	Urothelial papillary carcinoma G2 diagnosed
July 2015	Right hydronephrosis with multiple pulmonary metastases diagnosed
September 2015	Right sided “JJ” catheter placement
October 2015 to April 2016	Six cycles of gemcitabine/cisplatin chemotherapy
June 2016	Radical cystectomy, pelvic lymphadenectomy, left ureterocutaneous anastomosis, and right nephrectomy
June 2016	Pathohistology: urothelial carcinoma of the right ureter G2 pT2 N0 and urothelial carcinoma of the urinary bladder G2 pT1 N0
February 2017	Pulmonary disease progression
April 2017	SAUL study enrolment
May 2017	First atezolizumab (Tecentriq^®^)1200 mg i.v. q3w applied
May 2017	Development of vitiligo periorally, on hands, legs, and the scalp
July 2018	Right temporal region lipoma removal
February 2019	Stereotactic body radiation therapy of 55 Gy for a solitary pulmonary metastasis
April 2019	The 32nd atezolizumab (Tecentriq^®^) cycle

**Figure 1:**
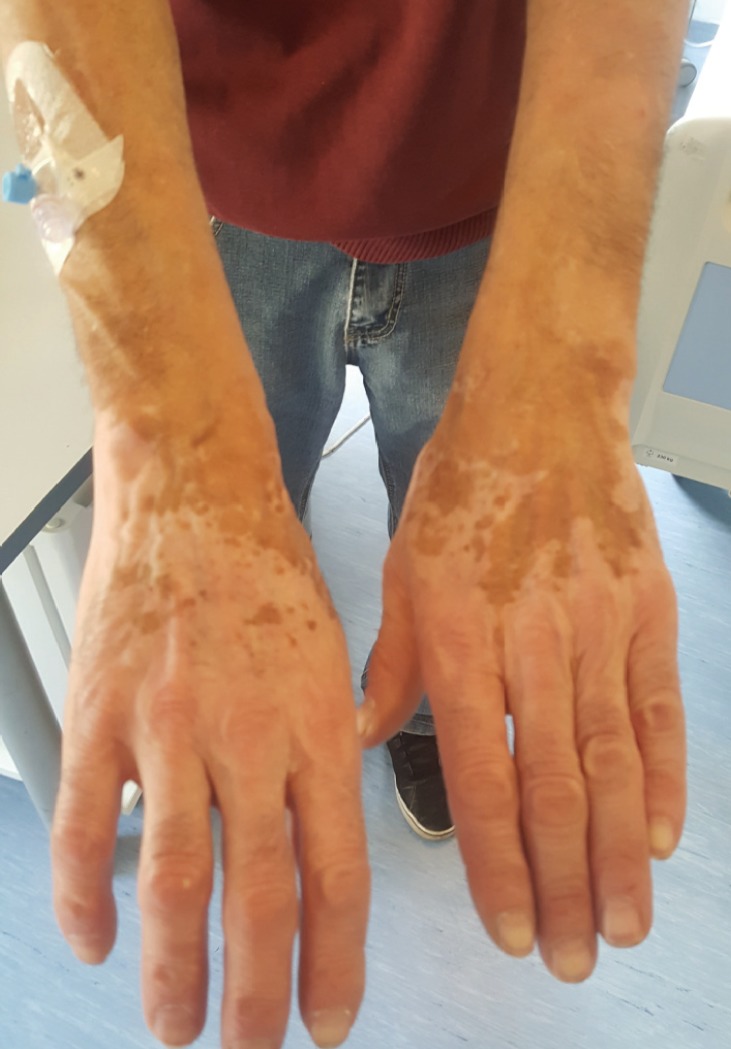
Vitiligo on patient’s hands.

**Figure 2:**
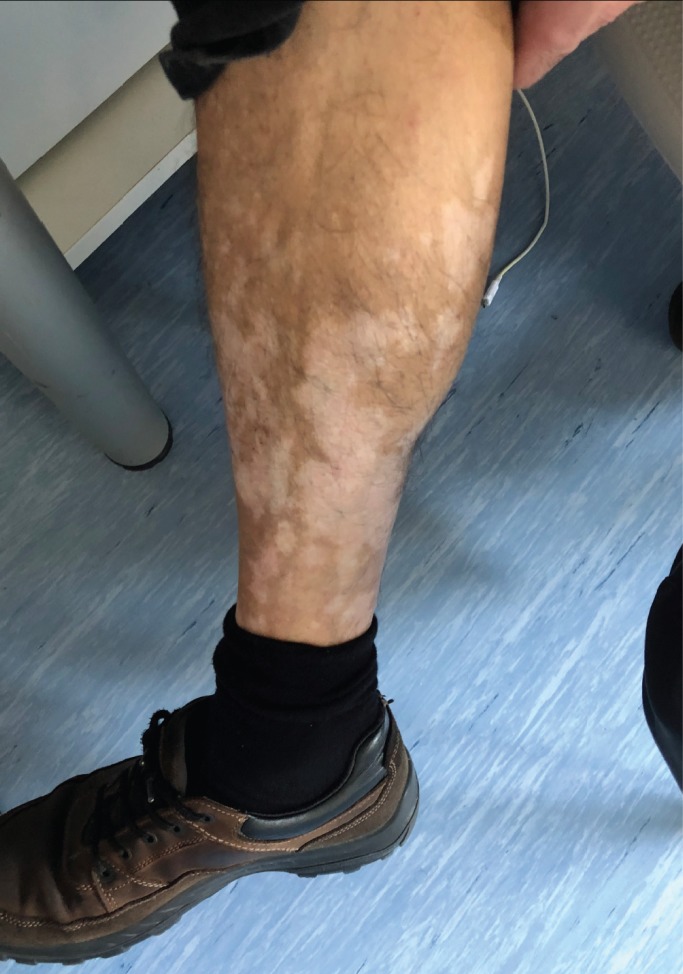
Vitiligo on patient’s lower leg.

In June 2016, the patient underwent radical cystectomy with pelvic lymphadenectomy, left ureterocutaneous anastomosis, and right nephrectomy. Pathohistology revealed two tumors: urothelial carcinoma of the right ureter, G2 pT2 N0, and urothelial carcinoma of the urinary bladder, G2 pT1 N0, measuring 1 × 2 cm and 11 × 9 cm, respectively (pN = 0/16, R0). In February 2017, pulmonary disease progression was noted. In April 2017, he was enrolled into the SAUL study and in May 2017 first received atezolizumab (Tecentriq^®^; Hoffmann-La Roche AG, Basel, Switzerland) 1200 mg i.v. q3w (7). Over the following 3 months, the two metastatic pulmonary lesions were considerably reduced in size, from 21 and 14 mm to 7 and 4 mm, respectively. Following the first atezolizumab administration in May 2017, the patient noted vitiligo periorally, on his hands (Figure 1), legs (Figure 2), and the scalp. He also experienced G2 fatigue and transient hypothyroidism. In July 2018, he noted a soft-tissue growth in the right temporal region, which was pathologically confirmed to be a lipoma.

Control MSCT scan in February 2019 showed that a solitary pulmonary metastasis grew from 7 to 15.25 mm, which is why stereotactic body radiation therapy of 55 Gy was applied. In April 2019, the 32nd atezolizumab cycle was administered. Now, 45 months after the diagnosis, the patient is 65 years old, with ECOG = 0. He responded to the first-line treatment with gemcitabine and cisplatin, which lasted for 16 months. His response to second-line atezolizumab therapy, with concomitant development of vitiligo as an immune related adverse event (irAE), still continues after 26 months of treatment.

## DISCUSSION

A few smaller clinical studies suggested that dermatological immune related adverse events following the administration of checkpoint inhibitors can be associated with treatment efficacy (8). Khan et al demonstrated that patients who experienced low-grade dermatological irAEs had longer OS in IMvigor211 (*P* = 0.024; hazard ratio [HR] 0.66; 95% confidence interval [CI] 0.45-0.95) and IMvigor210 (*P* = 0.0023; HR 0.53; 95% CI 0.35-0.80) trials (9). In their analysis, polygenic risk for psoriasis was associated with an increased odds of skin irAEs (*P* = 0.002; OR 1.79; 95% CI 1.24-2.40), while high polygenic risks for vitiligo (*P *= 0.0016; HR 0.58; 95% CI 0.41-0.81) and psoriasis (*P *= 5.5 × 10^−5^; HR 0.50; 95% CI 0.36-0.70), as well as low for atopic dermatitis (*P *= 0.0008; HR 0.57; 95% CI 0.41-0.79) were associated with longer OS under anti-PD-L1 atezolizumab monotherapy in comparison with chemotherapy (9).

Our patient’s OS of >23 months on atezolizumab in the second-line therapy is considerably higher than the median OS in the SAUL study of 8.7 months (IMvigor211-like patients OS 10.0 months) and, in our opinion, it highlights that concomitant skin autoimmunity can be associated with increased efficacy of atezolizumab in the treatment of metastatic urothelial carcinoma (7,10).

In conclusion, a positive association between overall survival and skin irAEs (vitiligo and psoriasis) reflects the known Th17 polarization of these diseases, indicating that individuals with high Th17 polarization benefit from PD-1 checkpoint blockade in the therapy of metastatic urothelial carcinoma. Genetic background and tumor factors likely interact and affect survival benefit associated with checkpoint blockade as compared with chemotherapy. Median OS in the second-line with classical chemotherapy for metastatic urothelial carcinoma is 6-9 months (10). Our patient remains asymptomatic with ECOG = 0 and an ongoing response to atezolizumab therapy for more than 26 months.
